# NICE guidance, eating disorders and older people

**DOI:** 10.1192/bjo.2026.11006

**Published:** 2026-04-29

**Authors:** Suzanne Heywood-Everett, Louisa J. Shirley, Noor Aqsa, Kate Fitzgerald, Ryan Horsfall

**Affiliations:** Primary Care Wellbeing Service, Bradford District Care NHS Foundation Trust, Bradford, UK; Division of Psychology and Mental Health, University of Manchester, UK; School of Psychology, University of Leeds, Leeds, UK

**Keywords:** Eating disorders, NICE guidance, older people

## Abstract

**Background:**

The treatment of eating disorders by the National Health Service in England and Wales adheres to the National Institute for Health and Care Excellence (NICE) guidelines, which were informed by an experimental evidence base.

**Aims:**

As the presentation and treatment of eating disorders have been shown to vary on the basis of age, we aimed to review the representation of older people in the evidence base for the NICE guidelines.

**Method:**

The evidence base was reviewed by identification of participant ages and the recruitment methods used in the experimental literature. The use of clinician referral was of particular interest, owing to the age-dependent risk of eating disorders being misdiagnosed in older adults.

**Results:**

The results highlighted low participant ages across the evidence base, most notably in anorexia nervosa samples. In accordance with the age data, a high frequency of clinician referral was used to recruit participants, with the highest rate identified in anorexia nervosa samples.

**Conclusions:**

NICE guidance fails to consider the economic, social, political and health contexts around onset or chronological development of an eating disorder, with no reference to comorbidities which are commonly reported with older people or how this might affect diagnosis, formulation and treatment recommendations. Research is urgently required to inform clinical recommendations for older adults.

Older adults with eating disorders remain markedly underrecognised in both research and national clinical guidelines, despite clear evidence of elevated mortality^1^ and morbidity and evidence that outcomes are significantly worse when the duration of untreated illness is prolonged.^2^ Anorexia nervosa has the highest prevalence among eating disorders in older people and the highest mortality rate of all psychiatric disorders^3^ across all ages. A range of medical, cultural, and historical factors complicate the recognition and treatment of eating disorders in later life.^4,5^ These include the stigma and shame of experiencing what is still perceived as a ‘youth-centric’ condition, as well as a persistent lack of robust data on eating disorders in midlife and older adults.^6^

Treatment approaches should consider age-adapted care tailored to older women and men, addressing the contextual pressures of midlife and ageing.^4^ These include sociocultural and political influences on body image, as well as the growing normalisation of aesthetic interventions such as cosmetic surgery, weight loss injections and anti-ageing treatments, which can exacerbate body dissatisfaction in later life.^4,7^ However, the establishment of data to inform services around a recommended care pathway for older people is still in its infancy. One recent systematic review of treatment outcomes for older people with eating disorders highlighted significant inconsistences among treatment packages for older people and concluded that the quality of case reports to date made it difficult to suggest specific assessment or treatment guidelines for this population.^8^

## Prevalence and diagnostic challenges

Although up to 1.2 million people in the UK are estimated to experience eating disorders, the prevalence in older adults remains unclear and underresearched. Unsurprisingly, widescale self-report data across England^9,10^ reveal substantially higher prevalence rates of eating disorder symptoms in older adults compared with analysis of diagnosis rates of eating disorders in primary care electronic health records,^11^ with both methods highlighting reduced rates of eating disorders in older females and males in comparison with their younger counterparts. Available estimated prevalence rates of eating disorders in older adults in Western samples range between 2.1 and 7.7% for older females and less than 1% for older males,^4^ although these are expected to be underestimates,^8^ particularly for males.^12^

Those with longstanding, untreated chronic eating disorders are likely to also have developed further physical health problems over their lifespan.^13^ Older people with eating disorders can experience a recurrence of an eating disorder later in life, experience a new onset later in life, or have a longstanding chronic problem from a much earlier age.^14^ Recent cross-sectional research has found that estimated prevalence rates of eating disorders in older people are far from negligible.^7^ This unmet need for age-sensitive intervention highlights the urgent need for clinicians and researchers to focus specifically on the unique differences of this population with respect to screening, assessment and treatment.

Prevalence data and systematic reviews of eating disorders in older populations have been significantly limited by historical and ongoing diagnostic bias. Diagnostic criteria have traditionally been developed from studies involving adolescents and young adults, often excluding atypical or later-onset presentations. Although anorexia nervosa has been included in classification systems since DSM-I, other major conditions such as bulimia nervosa and eating disorder not otherwise specified were only introduced in DSM-III during the 1980s. Consequently, many older individuals with subthreshold or atypical presentations, particularly those without hallmark features such as body image distortion or vomiting, may have gone undiagnosed for decades despite substantial distress or impairment.^15^

Clinical manifestations of eating disorders in later life may differ substantially from those in younger cohorts. Research has shown that older individuals with eating disorders are less likely to engage in vomiting but more likely to use laxatives for purging.^16^ Furthermore, a higher prevalence of current or prior obesity has been reported in this group, suggesting that later-life presentations may be shaped by a different trajectory of weight and body-related experiences. Given these distinctions, it is critical that health professionals working with older adults remain alert to the possibility of an eating disorder in individuals presenting with unexplained weight change, altered eating behaviours or new-onset mental health symptoms.^17^

## NICE guidelines on eating disorders

The clinical guidelines of the National Institute for Health and Care Excellence (NICE) provide the gold standard of research to which clinical practice in the National Health Service (NHS) adheres and by which it is measured England and Wales. Despite the assertion that NICE guideline NG69^18^ applies to individuals of all ages, it categorises people as ‘children’ (aged 0–12 years), ‘young people’ (13–17) or ‘adults’ (18+), with no specific guidance for ‘older adults’ or any consideration of age-related comorbid presentation, risk or treatment needs. In practice, this creates a one-size-fits-all model that neglects lifespan-specific factors. Definitions of eating disorders referred to here and routinely used in clinical services and in research are drawn from two similar diagnostic systems, DSM-5^19^ and ICD-11.^20^ One relevant difference in the DSM-5 is the inclusion of subjective binges in the definition of bulimia nervosa and binge-eating disorder (BED). Critically, eating disorders vary according to age group in terms of symptom presentation (for a review, see ref. ^17^), causal factors,^21^ treatment efficacy^6^ and mortality.^22^ Although full-threshold anorexia nervosa, bulimia nervosa and BED receive most attention, other specified feeding or eating disorder is the most prevalent eating disorder category overall, including in older populations, yet it remains marginalised in both research and guidelines.

Importantly, NICE can only make recommendations based on the evidence available at the time of guideline development; however, it can play an important leadership part by explicitly identifying evidence gaps. Eating disorders at all ages have become national, regional and local issues in the UK in response to the NHS Long Term Plan^23^ and The Community Mental Health Transformation Framework.^24^ National investment has led to the development of age-specific early-intervention services such as FREED (First Episode Rapid Early Intervention for Eating Disorders), which targeted at individuals aged 16–25 years. However, there is currently no equivalent model for older adults, despite their high risk and unique needs. By contrast, fewer than half of patients with eating disorders recover, and 20% develop longstanding eating disorder(s) (L-ED).^25^ This patient group is often complex, with extended treatment histories, multiple hospital admissions, a poor quality of life and the highest mortality rate of all.^25^ Several researchers have attempted to define or describe the group of patients experiencing L-ED using the concept of severe and enduring eating disorder.^25^ Although longstanding, severe and enduring eating disorder presentations often involve adults outside the age range targeted by early intervention services, existing protocols are rarely adapted for later life and may fail to address frailty, social isolation, multimorbidity or cognitive change.^17^ Furthermore, there are few examples in which NICE does refer specifically to age, and, where it does, it is either very general or refers to younger age bands only.

## Older adults, eating disorders and comorbidities

Older adults with eating disorders face a compounded burden of risk due to the interaction between chronic disordered eating and age-related physical vulnerability. L-ED are associated with significant medical complications, including osteoporosis, cardiac arrhythmias and immune suppression.^5,13^ These physical health comorbidities are frequently observed in ageing populations but may be mistakenly attributed to ‘natural’ ageing, leading to delayed or missed diagnoses.^17,26^

BED is particularly relevant in older populations due to its strong association with obesity and obesity-related health conditions. These include heightened risks of cardiovascular disease, diabetes, and functional decline, issues that may also be mistakenly attributed to ageing. Unlike other eating disorders, BED is often overlooked because it tends to co-occur with being overweight or obese, which may mask the underlying psychopathology and delay diagnosis. Despite the clinical significance, current guidance offers limited age-specific recommendations. The cumulative burden of physical and psychiatric comorbidities, as well as the associated healthcare costs, reinforces the importance of recognising older adults as a distinct clinical group.

## Older adult representation in the NICE guidelines on eating disorders

The risk of underdiagnosis^8^ and misdiagnosis of eating disorders in older adults has been highlighted,^6,15,26^ and it has been reported that a medical explanation of symptoms is more likely to be considered and investigated than body shape and eating issues in someone ‘over a certain age’.^27^ By emphasising age-specific risk factors and symptoms predominantly seen in younger populations and omitting references to possible differences and risks associated with older adults, the NICE guidelines inadvertently overlook the unique presentations and needs of older adults.

Further issues arise with respect to the applicability of the NICE evidence base for eating disorders when we consider the underrepresentation of older adults in clinical trials. Recruitment and retention challenges are well documented; these include age-related exclusion criteria, clinician gatekeeping, poor accessibility (e.g. mobility, transport, cost) and ineffective recruitment strategies such as digital-only campaigns or generic flyers.^28^ A recent UK study exploring older adults’ beliefs and attitudes towards eating disorders and help-seeking^29^ found that participants perceived a lack of information tailored to their age group, with many reporting that existing resources felt targeted at younger people. Notably, they suggested improvements such as more relevant web content and digital self-help but also highlighted the need for accessible offline options. This indicates that recruitment and engagement strategies must span both digital and traditional methods, reflecting the diverse preferences and access needs of older populations. Furthermore, stigma and internalised ageism further reduce the likelihood of help-seeking and research participation.

## The current work

This review examined the NICE NG69 guidelines on eating disorders with a focus on their applicability to midlife and older adults, a group consistently underrepresented in research, clinical pathways and national policy.^5^ As outlined above, cultural biases in diagnostic frameworks and assumptions about age-typical presentations continue to obscure the detection and treatment of eating disorders in later life. Although NICE NG69 claims to be inclusive of all ages, this paper interrogates the extent to which that claim is realised.

This study was initially planned as an audit, that is, ‘a quality improvement process that seeks to improve patient care and outcomes through a systematic review against explicit criteria and the implementation of change’.^30^ However, it immediately became apparent that when we applied the expected standards – particularly regarding age-inclusion criteria – to the evidence base, only a handful of studies could be considered. Consequently, the guidelines were reviewed to look at the age range in each of the studies used as evidence and the recruitment processes for the interventions being investigated.

## Method

### Interrogation of literature

Of the 200 papers in the NICE guideline evidence base, 115 were identified as experimental. Each experimental paper was scrutinised by examination of the mean age and age range of the sample, the recruitment method used to obtain participants, and the treatment options offered to these participants. In one paper, the mean age reported in the NICE guidelines did not correspond to details in the paper. Furthermore, the eating disorder diagnoses of the sample reported in the paper did not match that reported in the NICE guidelines; the data for this paper were therefore excluded from our analysis.

## Results

Each of the studies included in the guidelines involved participants with one of three eating disorders: anorexia nervosa, bulimia nervosa and BED. The distribution of studies across the three disorders was uneven, with BED samples comprising more than 48% of the total experimental studies, whereas anorexia nervosa samples accounted for less than 20%. Crucially, a substantial number of studies (nearly 50%) failed to report the age range of their participant sample: 10 of the 22 studies with an anorexia nervosa sample, 19 of 37 bulimia nervosa studies and 27 of 55 BED studies did not report the age range.

**Fig. 1:**
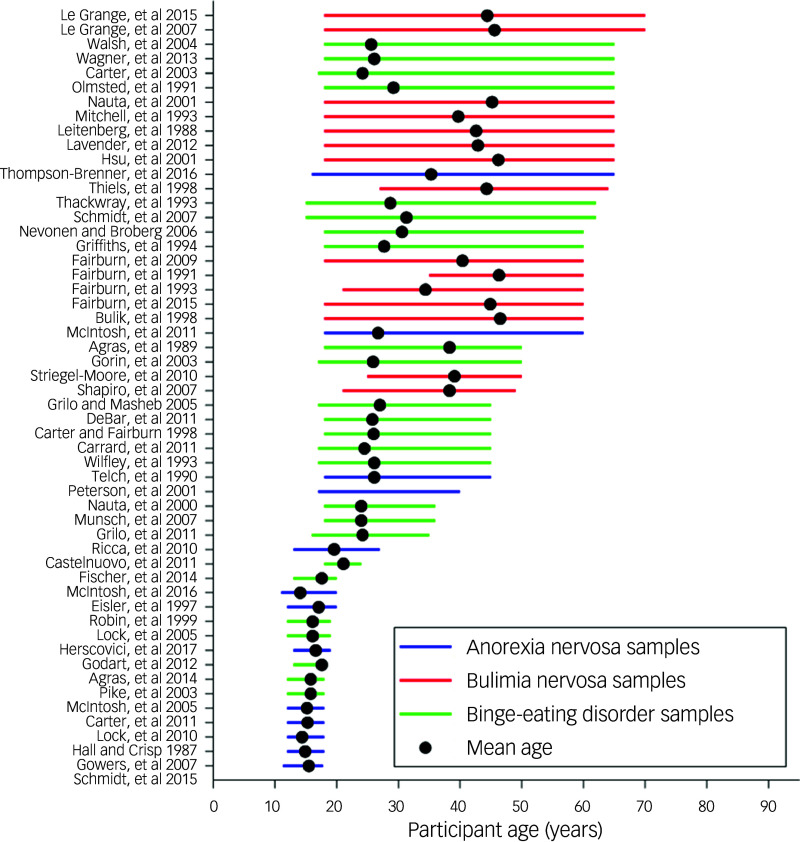
Mean age (black dots) and age ranges (coloured lines) of the participants involved in the experimental research cited in the National Institute for Health and Care Excellence guidelines for eating disorders. The colour of each line represents the diagnosed eating disorder for each participant sample. Note that experimental samples for which the age range or mean age was unreported were excluded from this figure. The references cited in this figure are available in the Supplementary material available at https://doi.org/10.1192/bjo.2026.11006.

The mean age of the samples across studies was calculated, as well as the mean of the maximum reported age across each study (Table 1). The overall mean age was lower in the anorexia nervosa and BED samples and notably higher in the bulimia nervosa samples. The average maximum age across each sample (when reported) painted a similar picture. Critically, there was not a single participant over the age of 65 years in either the anorexia nervosa or BED samples, and no participant over the age of 70 years in the bulimia nervosa samples (Fig. 1).

**Table 1:** Mean (standard deviation) age and average maximum age of the participant sample across the 115 experimental studies cited in the National Institute for Health and Care Excellence guidelines on eating disorders

Disorder studied	*N*	Mean age across studies, years (s.d.)	Mean of the reported maximum age in years across studies (s.d.)
Anorexia nervosa	22	21.94 (8.82)	26.73 (13.99)
Bulimia nervosa	37	41.66 (9.02)	61.56 (6.27)
Binge eating disorder	55	25.56 (4.44)	44.25 (17.56)
All studies	114	30.16 (10.78)	46.00 (18.75)

The recruitment method each study used is shown in Table 2. The recruitment strategies employed suggest that older adults face barriers to eating disorder research participation. Of note, 90.91% of the anorexia nervosa samples and 72.73% of the BED samples were recruited through clinician referral, although this was much lower for the bulimia nervosa samples (29.73%). Importantly, for the anorexia nervosa samples, clinician referral was the sole recruitment strategy in 77.27% of studies. This was much lower for bulimia nervosa and BED (13.51 and 43.64%, respectively). Furthermore, whereas 64.86% of bulimia nervosa studies and 35.71% of BED studies recruited participants through newspaper advertisements, there was just a single anorexia nervosa study which recruited using this older adult-friendly method. Some studies used multiple recruitment methods. Likewise, some studies recruited participants through methods not covered in this review. In total, three experimental studies failed to report their method of recruitment.

**Table 2:** Recruitment methods used in the experimental studies which informed the National Institute for Health and Care Excellence guidelines. Each value reflects the number of studies which used the stated sampling method. Note that some studies involved multiple recruitment methods, and therefore are represented in multiple columns

Disorder studied	Clinician referral (% of total), *n* (%)	Uni hospital referral (% of total), *n* (%)	Social media ad (% of total), *n* (%)	Newspaper ad (% of total), *n* (%)
Anorexia nervosa	21 (29)	2 (∼33)	1 (9)	1 (2)
Bulimia nervosa	11 (15)	2 (∼33)	3 (27)	24 (53)
Binge eating disorder	40 (56)	2 (∼33)	7 (64)	20 (45)
All studies	72	6	11	45

Uni, university; ad, advertisement.

## Discussion

In the current study, we examined integrated NICE guidelines for treatment of eating disorders with respect to their consideration of age, specifically older age adults. The specific eating disorders identified within these guidelines were limited to anorexia nervosa, BED and bulimia nervosa. The results highlighted notably low participant ages across the evidence base, as well as common use of clinician referral as a recruitment method, which would have screened out older people whose eating disorder had not been recognised or formally diagnosed. Further, we identified a lack of reporting of basic participant information in the eating disorder literature. These findings seriously question the applicability of the NICE guidelines to the treatment of eating disorders in older adults and highlight broader methodological issues within this evidence base.

### Lack of representation of older adults in the NICE guidelines

As noted above, the delayed recognition of eating disorders beyond anorexia nervosa by diagnostic frameworks has had lasting consequences: many individuals now in mid- or later life may have lived with undiagnosed, untreated illness for decades. These structural legacies continue to shape who is counted and cared for in research and practice. The findings of this review indicate that older adults are systematically excluded from the evidence base underpinning NICE guideline NG69 on eating disorders. Across 111 experimental studies, not a single participant over the age of 70 years was identified, and the inclusion of individuals aged 65 years and over was vanishingly rare; in addition, the mean ages of inclusion were remarkably low (*n* = 2; Fig. 1). This is particularly concerning given the recognised morbidity and mortality associated with eating disorders in later life.^5^ It has been argued that there is very little by way of an evidence base for interventions for older people with eating disorders,^15^ and so it is perhaps unsurprising that this is reflected in the NICE guidelines. As eating disorders vary based on age, in terms of both cause^21^ and prognosis,^6,22^ the low participant ages seen across a large amount of the research informing the NICE guideline raises significant issues around the claims of the guideline to be for ‘all ages’. Furthermore, mean ages were notably lower for research concerning anorexia nervosa in comparison with BED and, particularly, bulimia nervosa. As anorexia nervosa is the most common eating disorder reported in the literature on older adults,^5,15^ the low ages in these samples are of particular concern.

To ensure equitable healthcare for all individuals with eating disorders, it is imperative that NICE guidelines explicitly include the specific needs, presentations and service pathways relevant to older adults. Although high-level empirical evidence (level A) may be lacking owing to underrepresentation in trials, the use of level B and C evidence drawn from expert consensus, clinical case series and observational data is a legitimate and necessary approach in developing guidance for underresearched populations. Failure to do so perpetuates structural exclusion and compromises the principle of parity in mental health.

### Basic participant information is missing from large portions of the evidence base

The absence of transparent reporting of participant age data in nearly half the studies further undermines the applicability of guideline recommendations. This lack of demographic clarity compromises clinicians’ ability to assess generalisability^31^ and perpetuates a research landscape in which the needs of older adults are rendered invisible. A growing body of evidence suggests that eating disorders have been historically stereotyped as conditions that primarily affect young, White, cisgender women. This narrow framing contributes to significant underdiagnosis and undertreatment in older adults, men, people of colour and LGBTQ+ individuals, especially where intersecting identities exist.^32^ Emerging data also highlight the need to understand how ethnicity, cultural norms and socioeconomic status influence symptom expression, help-seeking and access to care in later life.^33^ To move towards equity, it is imperative that guideline development bodies and funders address these omissions and adopt inclusive recruitment and reporting standards that reflect the UK’s increasingly diverse ageing population.

### Ageing, complexity and clinical need

Older adults with eating disorders typically present with multifactorial complexity, including comorbid physical illnesses^13^ and psychiatric comorbidities.^16^ These intersecting domains necessitate integrated multidisciplinary care planning. Yet NG69 offers no guidance on the management of such comorbidities in this age group, nor does it reference conditions highly relevant to older adults, such as dementia or sensory impairments, which may affect diagnosis, treatment engagement and outcomes.

Recent reviews of the epidemiology and treatment of eating disorders in middle-aged and older adults have highlighted midlife as a critical period for both women and men for emergence of an eating disorder, with peaks during adolescence and around the age of 50 years.^4^ For women, the menopausal transition has been identified as a significant vulnerability window, with severe menopausal symptoms associated with greater eating disorder pathology. Similarly, hormonal disturbances in men during midlife have been proposed as potential contributors to disordered eating, although empirical evidence remains limited.

However, interpretation of these findings is complicated by the paucity of robust, population-level data on eating disorders in midlife and later life. Underrecognition, diagnostic bias and sampling issues contribute to systematic underestimation of prevalence and hinder the development of tailored interventions.^15^ Consequently, treatment approaches for older adults must be informed by emerging evidence, while the limitations of the current literature and the need for age- and gender-specific research are acknowledged.

Importantly, restrictive behaviours in older adults may be culturally or developmentally framed, with individuals expressing disordered eating through idioms such as ‘not needing much food’ or identity-laden constructs like ‘being tiny’ or ‘delicate’.^5,34^ This requires culturally and historically attuned clinical interpretation, which is entirely absent from current guidance.

### Recruitment methods and diagnostic pathways

Older adults are less likely to receive a timely diagnosis owing to diagnostic overshadowing, age-related stereotyping and medical misattribution.^15,26^ The fact that clinician referral was the dominant recruitment method across the NG69 evidence base is of particular concern, as this method systematically disadvantages older adults. Alternative recruitment methods, such as recruitment via general practitioners, registry outreach, community networks and print media, are more successful in reaching older populations.^28^ Yet such methods were infrequently used in the studies informing NG69, with newspaper advertising used in just one anorexia nervosa study, restricting the inclusivity of research samples.

### Structural gaps in services and national strategy

Despite the demographic ageing of the UK population and growing recognition of eating disorders as conditions affecting all age groups,^34^ there remains no national age-adapted care pathway for older adults with eating disorders. Many specialist eating disorder services, before the Community Mental Health Framework,^24^ historically sat within working-age adult NHS services, reinforcing diagnostic beliefs that this is a ‘young person’s illness’ and creating barriers to access, as well as barriers to training for staff.^34^ In contrast to models such as FREED for early intervention in youth,^35^ older adults may experience numerous barriers to access, including misdiagnosis, diagnostic delay, disjointed transitions between working-age and older adult services, and a lack of age-specific training among clinicians. All of these contribute to the duration of untreated illness; this can be further complicated by clinicians not considering eating disorders as a potential diagnosis in older people, as well as older people with eating disorders internalising messages that eating disorders are a ‘young woman’s problem’.^34^ NG69 fails to address this gap or provide any recommendation on adapting service models across the lifespan.

### Economic and public health implications

The failure to recognise and treat eating disorders in older adults carries profound economic and public health consequences. When left undiagnosed or untreated, these conditions contribute to escalating healthcare costs through delayed identification, emergency admissions, complex medical comorbidities and prolonged social care needs.^36^ These costs are likely to be underestimated in national estimates, which rarely stratify data by age or disaggregate costs for midlife and older adults, and they are very unlikely to be correlated with recorded eating disorder diagnoses. Recent UK analyses have highlighted the significant financial burden associated with eating disorders, and it has been proposed that early identification and intervention could lead to measurable reductions in both direct healthcare expenditure and indirect social care costs.^36,37^

### Diagnostic limitations and treatment opportunities

Diagnostic criteria for eating disorders, particularly anorexia nervosa, have historically been constructed around adolescent presentations. For example, the inclusion of amenorrhoea as a diagnostic criterion in DSM-IV effectively excluded postmenopausal individuals, reflecting a narrow developmental lens. Although this criterion was removed in DSM-5, many of the underlying assumptions about age and eating disorders persist in clinical practice and service design. In terms of screening, tools such as the SCOFF questionnaire, although validated in general populations,^38^ have only limited evidence supporting their use with older adults. Nonetheless, this questionnaire may have utility as a pragmatic first-line screener, particularly given the current lack of any age-adapted tools in this area.

The literature also highlights underrecognition of eating disorders in older men, who may present with non-classical symptoms and encounter greater stigma.^15^ Future diagnostic tools and research methodologies must therefore account for life-course diversity in terms of both gender and age.

Adapted cognitive–behavioural therapy for very old adults with eating disorders has shown promising outcomes, owing to its adaptability, structured nature and present-focused goals.^39^ Case literature and clinical experience suggest that structured cognitive–behavioural approaches (including cognitive–behavioural therapy for eating disorders) can be acceptable and beneficial in later life when adapted for medical risk, multimorbidity and frailty; however, delayed recognition and reluctance to seek help frequently limit access to effective treatment. These findings counter assumptions of treatment futility in later life; delays in recognition and reluctance to seek help often reduce opportunities for timely intervention, strengthening the case for more funded research and age-adapted pathways. These findings challenge longstanding assumptions that age is a barrier to effective psychological intervention. However, given the high prevalence of comorbid physical and mental health conditions, as well as increased social care needs in this population, psychological treatment often needs to be embedded within a broader multidisciplinary care framework. Diagnostic challenges, limited staff training in recognising eating disorders in later life, and a lack of robust, age-specific evidence further complicate service provision. In such contexts, drawing on other well-established clinical models may be necessary. Collaborative care, for example, has demonstrated effectiveness in treating depression among older adults, particularly in the context of mental–physical multimorbidity. It has also been associated with improved quality of life and cost-effectiveness through reduced hospital admissions. Despite this, research and service development applying collaborative care models to eating disorders has focused predominantly on younger populations. There is a pressing need for further evidence and policy guidance to address the complex and often high-risk biopsychosocial profiles of older adults with eating disorders especially those with L-ED or severe and enduring eating disorder.

Importantly, avoidant and restrictive food intake disorder (ARFID), a restrictive eating disorder not driven by concerns about weight or shape, was only formally recognised with the publication of DSM-5. ARFID is currently not included in the NICE guideline for eating disorders (NG69), yet it is associated with significant medical, psychological and social risks, many of which overlap with other eating disorders. Although ARFID is often identified in younger populations, it is increasingly recognised in older adults. In later life, presentations may include restrictive eating driven by fears of choking, gastrointestinal discomfort or sensory sensitivities. However, such symptoms are frequently misattributed to the normal ageing process or to physical health decline, resulting in underrecognition and missed opportunities for intervention. This diagnostic oversight could contribute to deteriorating health outcomes. The *NHS England Framework for Good Practice in Delivering Support to Adults and Older Adults with ARFID* underscores the importance of recognising ARFID in older populations.^40^ Increasing clinical awareness and embedding ARFID into staff training and multidisciplinary care planning are essential for improving detection and intervention in this group.^40^

### Implications and recommendations

Although written with the intention of raising awareness of the lack of inclusion of older adults in the diagnosis and treatment of eating disorders, this paper has wider implications for services, commissioners and clinicians who rely on NICE guidance to promote and fund research into detection and diagnostic indicators of eating disorders and the development of treatment approaches across the adult lifespan.

This review highlights critical shortcomings in the evidence base underpinning NICE guideline NG69, particularly regarding the representation, diagnosis and treatment of eating disorders in older adults. The dominance of clinician-referred, youth-centric studies limits generalisability and fails to account for the physiological, psychological, cultural and social complexities of later-life eating disorders. As NICE can only appraise the evidence available, targeted research and service evaluation are needed to inform future updates and to support equitable commissioning and care across the adult lifespan.

Box 1 Key recommendations.

- **NICE guideline NG69 should be updated to highlight** the evidence gap in later-life eating disorders, and this should be flagged as a priority area for commissioned, funded research – spanning identification, diagnostic indicators and effective treatments for older adults – so that future updates can be based on age-inclusive data.
- **Future research in eating disorders** should include transparent age-stratified reporting in trials and service evaluations (e.g. age range ≥65 years, outcomes, attrition, adverse events).
- **Positive inclusivity of older adults in trials:** remove upper-age cut-offs in studies and services and use recruitment and/or retention methods that work for older people (e.g. general practitioner and/or community routes, phone or post options, transport support – not digital-only access).
- **Build later-life early intervention:** develop and test early-intervention pathways for older adults (similar in principle to FREED) that explicitly address medical risk, multimorbidity and frailty.
- **Fund lifespan evidence:** commission research and service evaluations across the adult lifespan, including community-based care and digital options with offline alternatives (e.g. telephone, printed materials, in-person).
- **Train clinicians to identify later-life eating disorders:** provide specialist training on later-life presentations, differential diagnosis and diagnostic overshadowing to support earlier recognition and referral across mental health and physical health services.
- **Join up care pathways:** create integrated pathways linking eating disorder services with primary care, older people’s mental health, acute medical services and the voluntary sector (e.g. Age UK), with clear roles and escalation routes.

Finally, equitable and effective care for older adults with eating disorders will only be achieved through coordinated reform across research, policy and clinical practice, with NICE having a critical leadership role. As highlighted throughout this review, older adults with eating disorders face a double burden: clinical vulnerability due to high rates of medical and psychiatric comorbidity, and structural neglect within mental health systems for eating disorders largely designed around younger populations. With the ageing population growing rapidly and increasing recognition of eating disorders emerging or persisting into midlife and later life, the absence of targeted guidance risks widening health inequalities.

Current NICE guidance (NG69) does not sufficiently reflect the complexity of eating disorder presentations in older adults, including those with longstanding illness, late-onset presentations or comorbid conditions such as frailty, cognitive decline and chronic physical illness. This omission limits service design, constrains commissioning and undermines the ability of workforce initiatives such as all-age multisystem eating disorder clinical pathway expansion and community mental health transformation to deliver truly inclusive, age-responsive care. Revising NICE guidelines to explicitly highlight these needs would support the development of multidisciplinary, age-informed approaches that recognise the full biopsychosocial context of eating disorders in later life. Addressing these gaps is not only a matter of scientific and clinical rigour but of parity and justice in care for our ageing population.

## Supporting information

10.1192/bjo.2026.11006.sm001 Heywood-Everett et al. supplementary material Heywood-Everett et al. supplementary material

## Data Availability

The data that support the findings of this study are available on request from the corresponding author, S.H.-E.

## Supplementary material

The supplementary material is available online at https://doi.org/10.1192/bjo.2026.11006
