# Supplementary material for: NICE guidance, eating disorders and older people
Source: BJPsych Open. 2026 Apr 29;12(3):e123. doi: 10.1192/bjo.2026.11006 (PMC13126286; doi:10.1192/bjo.2026.11006)
Supplement: Heywood-Everett et al. supplementary material [file S2056472426110060sup001.docx]

**References included in Figure 1:**

Le Grange, D., Lock, J., Agras, W. S., Bryson, S. W., & Jo, B. (2015). Randomized clinical trial of family-based treatment and cognitive-behavioral therapy for adolescent bulimia nervosa. *Journal of the American Academy of Child & Adolescent Psychiatry*, *54*(11), 886-894.

Le Grange, D., Crosby, R. D., Rathouz, P. J., & Leventhal, B. L. (2007). A randomized controlled comparison of family-based treatment and supportive psychotherapy for adolescent bulimia nervosa. *Archives of General Psychiatry*, *64*(9), 1049-1056

Walsh, B. T., Kaplan, A. S., Attia, E., Olmsted, M., Parides, M., Carter, J. C. & Rockert, W. (2004). Fluoxetine after weight restoration in anorexia nervosa: a randomized controlled trial. *Jama*, *295*(22), 2605-2612.

Wagner, B., Nagl, M., Dölemeyer, R., Klinitzke, G., Steinig, J., Hilbert, A., & Kersting, A. (2013). Randomized controlled trial of an internet-based cognitive-behavioral treatment program for binge-eating disorder. *Behavior Therapy*, *47*(4), 500-514.

Carter, J. C., Olmsted, M. P., Kaplan, A. S., McCabe, R. E., Mills, J. S., & Aimé, A. (2003). Self-help for bulimia nervosa: a randomized controlled trial. *American Journal of Psychiatry*, *160*(5), 973-978.

Olmsted, M. P., Davis, R., Rockert, W., Irvine, M. J., Eagle, M., & Garner, D. M. (1991). Efficacy of a brief group psychoeducational intervention for bulimia nervosa. *Behaviour Research and Therapy*, *29*(1), 71-83.

Nauta, H., Hospers, H., & Jansen, A. (2001). One‐year follow‐up effects of two obesity treatments on psychological well‐being and weight. *British Journal of Health Psychology*, *6*(3), 271-284.

Mitchell, J. E., Pyle, R. L., Eckert, E. D., Zollman, M., Crosby, R., Zimmerman, R., ... & Seim, H. (1993). Cognitive‐behavioral group psychotherapy of bulimia nervosa: Importance of logistical variables. *International Journal of Eating Disorders*, *14*(3), 277-287.

Leitenberg, H., Rosen, J. C., Gross, J., Nudelman, S., & Vara, L. S. (1988). Exposure plus response-prevention treatment of bulimia nervosa. *Journal of Consulting and Clinical Psychology*, *56*(4), 535.

Lavender, A., Startup, H., Naumann, U., Samarawickrema, N., DeJong, H., Kenyon, M.,& Schmidt, U. (2012). Emotional and social mind training: a randomised controlled trial of a new group-based treatment for bulimia nervosa. *PLoS One*, *7*(10), e46047.

Hsu, L. K. G., Rand, W., Sullivan, S., Liu, D. W., Mulliken, B., McDonagh, B., & Kaye, W. H. (2001). Cognitive therapy, nutritional therapy and their combination in the treatment of bulimia nervosa. *Psychological medicine*, *31*(5), 871-879.

Thompson‐Brenner, H., Shingleton, R. M., Thompson, D. R., Satir, D. A., Richards, L. K., Pratt, E. M., & Barlow, D. H. (2016). Focused vs. Broad enhanced cognitive behavioral therapy for bulimia nervosa with comorbid borderline personality: A randomized controlled trial. *International Journal of Eating Disorders*, *49*(1), 36-49.

Thiels, C., Schmidt, U., Treasure, J., Garthe, R., & Troop, N. (1998). Guided self-change for bulimia nervosa incorporating use of a self-care manual. *American Journal of Psychiatry*, *155*(7), 947-953.

Thackwray, D. E., Smith, M. C., Bodfish, J. W., & Meyers, A. W. (1993). A comparison of behavioral and cognitive-behavioral interventions for bulimia nervosa. *Journal of consulting and clinical psychology*, *61*(4), 639.

Schmidt, U., Lee, S., Beecham, J., Perkins, S. J., Treasure, J., Yi, I., Dodge, E., Macdonald, P., Keville, S., Johnson-Sabine, E., Jenkins M., & Eisler, I. (2007). A randomized controlled trial of family therapy and cognitive behavior therapy guided self-care for adolescents with bulimia nervosa and related disorders. *American Journal of Psychiatry, 164*(4), 591–598.

Nevonen, L., & Broberg, A. G. (2006). A comparison of sequenced individual and group psychotherapy for patients with bulimia nervosa. *International Journal of Eating Disorders*, *39*(2), 117-127.

Griffiths, R. A., Hadzi‐Pavlovic, D., & Channon‐Little, L. (1994). A controlled evaluation of hypnobehavioural treatment for bulimia nervosa: Immediate pre‐post treatment effects. *European Eating Disorders Review*, *2*(4), 202-220.

Fairburn, C. G., Cooper D Phil, Dip Psych, Z., Doll D Phil, H. A., O’Connor, M. E., Bohn D Phil, Dip Psych, K., Hawker, D. M., ... & Palmer, R. L. (2009). Transdiagnostic cognitive-behavioral therapy for patients with eating disorders: a two-site trial with 60-week follow-up. *American Journal of Psychiatry*, *166*(3), 311-319.

Fairburn, C. G., Jones, R., Peveler, R. C., Carr, S. J., Solomon, R. A., O'Connor, M. E. & Hope, R. A. (1991). Three psychological treatments for bulimia nervosa: A comparative trial. *Archives of General Psychiatry*, *48*(5), 463-469.

Fairburn, C. G., Jones, R., Peveler, R. C., Hope, R. A., & O'Connor, M. (1993). Psychotherapy and bulimia nervosa: Longer-term effects of interpersonal psychotherapy, behavior therapy, and cognitive behavior therapy. *Archives of General Psychiatry*, *50*(6), 419-428.

Fairburn, C. G., Bailey-Straebler, S., Basden, S., Doll, H. A., Jones, R., Murphy, R., & Cooper, Z. (2015). A transdiagnostic comparison of enhanced cognitive behaviour therapy (CBT-E) and interpersonal psychotherapy in the treatment of eating disorders. *Behaviour Research and Therapy*, *70*, 64–71.

Bulik, C. M., Sullivan, P. F., Carter, F. A., McIntosh, V. V., & Joyce, P. R. (1998). The role of exposure with response prevention in the cognitive-behavioural therapy for bulimia nervosa. *Psychological Medicine*, *28*(3), 611-623.

McIntosh, V. V. W., Carter, F. A., Bulik, C. M., Frampton, C. M. A., & Joyce, P. R. (2011). Five-year outcome of cognitive behavioral therapy and exposure with response prevention for bulimia nervosa. *Psychological Medicine*, *41*(5), 1061-1071.

Agras, W. S., Schneider, J. A., Arnow, B., Raeburn, S. D., & Telch, C. F. (1989). Cognitive-behavioral and response-prevention treatments for bulimia nervosa. *Journal of consulting and clinical psychology*, *57*(2), 215.

Gorin, A. A., Le Grange, D., & Stone, A. A. (2003). Effectiveness of spouse involvement in cognitive behavioral therapy for binge eating disorder. *International Journal of Eating Disorders*, *33*(4), 421-433.

Striegel-Moore, R. H., Wilson, G. T., DeBar, L., Perrin, N., Lynch, F., Rosselli, F., & Kraemer, H. C. (2010). Cognitive behavioral guided self-help for the treatment of recurrent binge eating. *Journal of consulting and clinical psychology*, *78*(3), 312.

Shapiro, J. R., Reba‐Harrelson, L., Dymek‐Valentine, M., Woolson, S. L., Hamer, R. M., & Bulik, C. M. (2007). Feasibility and acceptability of CD‐ROM‐based cognitive‐behavioural treatment for binge‐eating disorder. *European Eating Disorders Review: The Professional Journal of the Eating Disorders Association*, *15*(3), 175-184.

Grilo, C. M., & Masheb, R. M. (2005). A randomized controlled comparison of guided self-help cognitive behavioral therapy and behavioral weight loss for binge eating disorder. *Behaviour Research and Therapy*, *43*(11), 1509-1525.

DeBar, L. L., Striegel-Moore, R. H., Wilson, G. T., Perrin, N., Yarborough, B. J., Dickerson, J. & Kraemer, H. C. (2011). Guided self-help treatment for recurrent binge eating: Replication and extension. *Psychiatric Services*, *62*(4), 367-373.

Carter, J. C., & Fairburn, C. G. (1998). Cognitive–behavioral self-help for binge eating disorder: A controlled effectiveness study. *Journal of consulting and clinical psychology*, *66*(4), 616.

Carrard, I., Crépin, C., Rouget, P., Lam, T., Golay, A., & Van der Linden, M. (2011). Randomised controlled trial of a guided self-help treatment on the Internet for binge eating disorder. *Behaviour research and therapy*, *49*(8), 482-491.

Wilfley, D. E., Agras, W. S., Telch, C. F., Rossiter, E. M., Schneider, J. A., Cole, A. G., & Raeburn, S. D. (1993). Group cognitive-behavioral therapy and group interpersonal psychotherapy for the nonpurging bulimic individual: a controlled comparison. *Journal of consulting and clinical psychology*, *61*(2), 296.

Telch, C. F., Agras, W. S., Rossiter, E. M., Wilfley, D., & Kenardy, J. (1990). Group cognitive-behavioral treatment for the nonpurging bulimic: an initial evaluation. *Journal of Consulting and Clinical Psychology*, *58*(5), 629.

Peterson, C. B., Mitchell, J. E., Engbloom, S., Nugent, S., Mussell, M. P., Crow, S. J., & Thuras, P. (2001). Self‐help versus therapist‐led group cognitive‐behavioral treatment of binge eating disorder at follow‐up. *International Journal of Eating Disorders*, *30*(4), 363-374.

Nauta, H., Hospers, H., Kok, G., & Jansen, A. (2000). A comparison between a cognitive and a behavioral treatment for obese binge eaters and obese non-binge eaters. *Behavior Therapy*, *31*(3), 441-461.

Munsch, S., Biedert, E., Meyer, A., Michael, T., Schlup, B., Tuch, A., & Margraf, J. (2007). A randomized comparison of cognitive behavioral therapy and behavioral weight loss treatment for overweight individuals with binge eating disorder. *International journal of eating disorders*, *40*(2), 102-113.

Grilo, C. M., Masheb, R. M., Wilson, G. T., Gueorguieva, R., & White, M. A. (2011). Cognitive–behavioral therapy, behavioral weight loss, and sequential treatment for obese patients with binge-eating disorder: A randomized controlled trial. *Journal of consulting and clinical psychology*, *79*(5), 675.

Ricca, V., Castellini, G., Mannucci, E., Sauro, C. L., Ravaldi, C., Rotella, C. M., & Faravelli, C. (2010). Comparison of individual and group cognitive behavioral therapy for binge eating disorder. A randomized, three-year follow-up study. *Appetite*, *55*(3), 656-665.

Castelnuovo, G., Manzoni, G. M., Villa, V., Cesa, G. L., & Molinari, E. (2011). Brief strategic therapy vs cognitive behavioral therapy for the inpatient and telephone-based outpatient treatment of binge eating disorder: the STRATOB randomized controlled clinical trial. *Clinical practice and epidemiology in mental health: CP & EMH*, *7*, 29.

Fischer, S., Meyer, A. H., Dremmel, D., Schlup, B., & Munsch, S. (2014). Short-term cognitive-behavioral therapy for binge eating disorder: long-term efficacy and predictors of long-term treatment success. *Behaviour Research and Therapy*, *58*, 36-42.

McIntosh, V. V., Jordan, J., Carter, J. D., Frampton, C. M., McKenzie, J. M., Latner, J. D., & Joyce, P. R. (2016). Psychotherapy for transdiagnostic binge eating: A randomized controlled trial of cognitive-behavioural therapy, appetite-focused cognitive-behavioural therapy, and schema therapy. *Psychiatry research*, *240*, 412-420.

Eisler, I., Dare, C., Russell, G. F., Szmukler, G., le Grange, D., & Dodge, E. (1997). Family and individual therapy in anorexia nervosa: A 5-year follow-up. *Archives of general psychiatry*, *54*(11), 1025-1030.

Robin, A. L., Siegel, P. T., Moye, A. W., Gilroy, M., Dennis, A. B., & Sikand, A. (1999). A controlled comparison of family versus individual therapy for adolescents with anorexia nervosa. *Journal of the American Academy of Child & Adolescent Psychiatry*, *38*(12), 1482-1489.

Lock, J., Agras, W. S., Bryson, S., & Kraemer, H. C. (2005). A comparison of short-and long-term family therapy for adolescent anorexia nervosa. *Journal of the American Academy of Child & Adolescent Psychiatry*, *44*(7), 632-639.

Herscovici, C. R., Kovalskys, I., & Orellana, L. (2017). An exploratory evaluation of the family meal intervention for adolescent anorexia nervosa. *Family Process*, *56*(2), 364–375.

Godart, N., Berthoz, S., Curt, F., Perdereau, F., Rein, Z., Wallier, J. & Jeammet, P. (2012). A randomized controlled trial of adjunctive family therapy and treatment as usual following inpatient treatment for anorexia nervosa adolescents. *PloS one*, *7*(1), e28249.

Agras, W. S., Lock, J., Brandt, H., Bryson, S. W., Dodge, E., Halmi, K. A. & Woodside, B. (2014). Comparison of 2 family therapies for adolescent anorexia nervosa: a randomized parallel trial. *JAMA psychiatry*, *71*(11), 1279-1286.

Pike, K. M., Walsh, B. T., Vitousek, K., Wilson, G. T., & Bauer, J. (2003). Cognitive behavior therapy in the posthospitalization treatment of anorexia nervosa. *American Journal of Psychiatry*, *160*(11), 2046-2049.

McIntosh, V. V., Jordan, J., Carter, F. A., Luty, S. E., McKenzie, J. M., Bulik, C. M., & Joyce, P. R. (2005). Three psychotherapies for anorexia nervosa: a randomized, controlled trial. *American Journal of Psychiatry*, *162*(4), 741-747.

Carter, F. A., Jordan, J., McIntosh, V. V., Luty, S. E., McKenzie, J. M., Frampton, C. M., & Joyce, P. R. (2011). The long‐term efficacy of three psychotherapies for anorexia nervosa: A randomized, controlled trial. *International Journal of eating disorders*, *44*(7), 647-654.

Lock, J., Le Grange, D., Agras, W. S., Moye, A., Bryson, S. W., & Jo, B. (2010). Randomized clinical trial comparing family-based treatment with adolescent-focused individual therapy for adolescents with anorexia nervosa. *Archives of general psychiatry*, *67*(10), 1025-1032.

Hall, A., & Crisp, A. H. (1987). Brief psychotherapy in the treatment of anorexia nervosa: outcome at one year. *The British Journal of Psychiatry*, *151*(2), 185-191.

Gowers, S. G., Clark, A., Roberts, C., Griffiths, A., Edwards, V., Bryan, C. & Barrett, B. (2007). Clinical effectiveness of treatments for anorexia nervosa in adolescents: randomised controlled trial. *The British Journal of Psychiatry*, *191*(5), 427-435.

Schmidt, U., Magill, N., Renwick, B., Keyes, A., Kenyon, M., Dejong, H.,& Landau, S. (2015). The Maudsley Outpatient Study of Treatments for Anorexia Nervosa and Related Conditions (MOSAIC): Comparison of the Maudsley Model of Anorexia Nervosa Treatment for Adults (MANTRA) with specialist supportive clinical management (SSCM) in outpatients with broadly defined anorexia nervosa: A randomized controlled trial. *Journal of consulting and clinical psychology*
